# Thyroid hormone receptor beta-2 (TRβ2) overexpression modulates photoreceptor phenotype diversity in a ligand-dependent manner

**DOI:** 10.3389/fcell.2025.1697930

**Published:** 2025-10-20

**Authors:** Emmanuel Owusu Poku, Matthew R. Fonte, Tyler J. Jensen, Sydney P. Inman, Robert D. Mackin, Deborah L. Stenkamp

**Affiliations:** ^1^ Department of Biological Sciences, University of Idaho, Moscow, ID, United States; ^2^ WWAMI Medical Education Program, University of Washington School of Medicine, Moscow, ID, United States

**Keywords:** retina, cone photoreceptor, thyroid hormone, zebrafish, nuclear hormone signaling, opsin, development, transcription factor

## Abstract

**Background:**

Vertebrate color vision results from the specification of photoreceptor subtypes expressing distinct opsins. Thyroid hormone (TH) and its receptor TRβ2 are essential regulators of long-wavelength-sensitive (LWS) cone development, but their ligand-dependent roles in regulating cone subtype fate remain unclear.

**Methods:**

We investigated how varying TH availability and TRβ2 overexpression impact cone photoreceptor diversity and opsin expression using a gain-of-function transgenic zebrafish line (*crx:trβ2*), which expresses TRβ2 in all photoreceptors, and manipulated TH levels through T3 supplementation or ablation of the thyroid gland. Samples were analyzed through a combination of hybridization chain reaction *in situ* hybridization, confocal microscopy, and quantitative RT-PCR.

**Results:**

we found evidence consistent with the hypothesis that unliganded TRβ2 predominantly promotes *lws2* expression, while liganded TRβ2 upregulates *lws1*, following a dose-dependent and temporally dynamic pattern. Overexpression of TRβ2 promoted co-expression of *lws2* in non-LWS cones, suggesting possible photoreceptor transfating. TH supplementation amplified these effects and induced changes in numbers and morphologies of cone subtypes with no obvious evidence of cell death. We also identified spatially distinct expression of key TH regulatory genes (*dio2*, *dio3b*, *mct8*) in the retina and retinal pigment epithelium, which responded dynamically to manipulation of TH levels.

**Conclusion:**

Our findings suggest that TRβ2 exerts differential effects on cone opsin expression depending on presence and quantity of ligand.

## 1 Introduction

Vertebrate color vision depends on opsin expression in cone photoreceptors. Ancestral vertebrate genomes harbored four cone opsin genes (*M/LWS, RH2, SWS1, SWS2*) and one rhodopsin gene (*RH1*); placental mammals lost the *SWS2* and *RH2* genes, resulting in dichromatic color vision in most mammals (Bowmaker, 2008; Davies et al., 2012). Some primates gained trichromacy via tandem duplication of the *LWS* gene, producing *LWS* and *MWS* genes, which are spectrally divergent, having long-vs. middle-wavelength sensitivities (Nathans et al., 1986; Hunt et al., 1998). Humans exhibit trichromatic vision through unique expression of distinct opsin genes in individual cones (Baden and Osorio, 2019).

In contrast, many fish genomes retain all ancestral opsins and have also undergone tandem duplications (Musilova et al., 2019), offering robust systems for studying opsin gene regulation. The zebrafish *lws* opsin genes share a common ancestral gene with human *LWS/MWS*, and are tandemly-duplicated, *lws1* and *lws2*, showing 93% sequence identity but distinct spectral sensitivities. Similarly, the zebrafish *RH2* opsin locus is a tandemly-quadruplicated array (*rh2-1, rh2-2, rh2-3, rh2-4*) (Chinen et al., 2003; Hofmann and Carleton, 2009).

Several models have been advanced to explain regulation of replicated opsin genes. In humans, a locus control region (LCR) upstream of *LWS* interacts with either gene in the array, promoting exclusive *LWS* or *MWS* expression in individual cones (Smallwood et al., 2002; Peng and Chen, 2011). A “stochastic” model attributes the average 3:1 LWS:MWS ratio to LCR proximity (Smallwood et al., 2002), although does not explain topographic gradients of this ratio (Hayashi et al., 1999). An alternative model involves spatiotemporal changes in LWS:MWS ratios during fetal development, with potential functions for retinoic acid (RA) signaling observed in human retinal organoids (Hadyniak et al., 2024). Zebrafish also display LWS1:LWS2 gradients, with *lws1* abundant peripherally and *lws2* centrally (Takechi and Kawamura, 2005; Ogawa and Corbo, 2021). We demonstrated that thyroid hormone (TH) regulates the *lws* opsin array in zebrafish, promoting *lws1* and inhibiting *lws2* expression to a greater extent than RA, and across the lifespan (Mitchell et al., 2015; Mackin et al., 2019; Farre et al., 2023b). TH also modulates expression of *sws1* and *sws2*, selectively influences *rh2* paralogs, and paralogs encoding gamma subunits of cone transducin (Mackin et al., 2019; Farre et al., 2023a). TH is known to bind TH receptors, which form homodimers or heterodimers with RXRs and interact with TH response elements (TREs) on DNA (Wärnmark et al., 2003). The TH receptor TRβ2, expressed in cones, is essential for LWS cone differentiation (Suzuki et al., 2013; Deveau et al., 2020; Volkov et al., 2020). RXRγ and TRβ2 are required to inhibit S-opsin (*SWS1*) expression in mice (Roberts et al., 2005; Roberts et al., 2006). Misexpression of *TRβ2* in mouse rods results in the complete absence of rhodopsin and upregulation of M-opsin (orthologous to LWS) (Ng et al., 2011).

Zebrafish with transient overexpression of *trβ2* display increased LWS cone density and reduced SWS1 cones (Suzuki et al., 2013). Photopic ERGs in stable germline transgenics show dominance of LWS2 cone functional responses at the expense of other cone types and the adults exhibit functional “red-dichromacy” (LWS1 and LWS2 only or predominantly) with heightened LWS1 responses (Nelson et al., 2022). Collectively, evidence suggests that TH and Trβ2 are key upstream regulators in the determination of long wavelength sensitive cone photoreceptor types, and we hypothesize TRβ2 may have been evolutionarily co-opted to also regulate *lws1* vs. *lws2* expression in zebrafish.

This study investigates the impact of hypothyroid, euthyroid, and hyperthyroid states together with over/ectopic expression of TRβ2 on opsin transcripts and selected TH homeostasis-related transcripts in larval zebrafish retina and RPE. Our results support the ‘transcriptional dominance model’ of photoreceptor cell differentiation—a photoreceptor precursor’s fate is determined by dominant transcription factors at a specific developmental stage (Swaroop et al., 2010). We demonstrate that ectopic *trβ2* expression enhances *lws2* expression in specific photoreceptor classes, in varying proportions. Further, we find support for the hypothesis that liganded Trβ2 enhances *lws1* expression, while unliganded Trβ2 favors *lws2* expression.

## 2 Materials and methods

### 2.1 Animals

Zebrafish (*Danio rerio*) were bred and maintained following Westerfield’s guidelines (Westerfield, 2007) in recirculating, monitored, and filtered water systems. They were kept on a 14-h light/10-h dark cycle at a steady temperature of 28.5 °C. All procedures were approved by the Institutional Animal Care and Use Committee (IACUC) at the University of Idaho. Wildtype (WT) zebrafish used in this study were originally obtained from Scientific Hatcheries or Aquatica Tropicals (strain is now available from Segrest Farms, Gibsonton, FL). To maintain transparency for whole-mount confocal microscopy, embryos were treated with 0.003% phenylthiourea (PTU) to inhibit melanin synthesis (Westerfield, 2007). The transgenic line Tg (*crx*:MYFP-2a-*trβ2*)^*q21tg*^ was generously provided by Rachel Wong (University of Washington), and contains *trβ2* and MYFP coding sequences linked by a 2A peptide, driven by the *crx* promoter (Nelson et al., 2022). This line features over- and ectopic expression of *trβ2* in all photoreceptors and a subset of bipolar cells. The transgenic strain *Tg (tg:nVenus-2a-nfnB)* ^*wp.rt8*^, kindly provided by David Parichy (University of Virginia), expresses nuclear Venus (a YFP) and the bacterial nitroreductase nfnB, driven by the thyroglobulin promoter allowing metronidazole-mediated ablation of thyroid follicular cells (McMenamin et al., 2014; Mackin et al., 2019). The *trβ* CRISPR mutant (*thrb* ^*stl6 2*^) was a generous gift from Joseph Corbo (Washington University in St. Louis) (Volkov et al., 2020).

### 2.2 Genotyping

Genomic DNA was isolated from larval zebrafish for PCR-based genotyping of the *thrb* mutants. Posterior body segments were incubated in 50 μL of 50 mM NaOH at 95 °C for 10 min, cooled to 4 °C, and neutralized with 5 μL of 1 M Tris (pH 8.0). DNA extracts were then diluted 1:10 in nuclease-free water prior to PCR. The primer sequences used were identical to those previously described by (Volkov et al., 2020).

### 2.3 Thyroid hormone and metronidazole treatments

Triiodothyronine (T3; Sigma) stock solutions were prepared in dimethyl sulfoxide (DMSO) and stored at −20 °C in the dark. For larval experiments, final T3 concentrations (4, 20, 100, and 500 nM) were achieved by adding 1,000 x T3 to system water, maintaining DMSO at 0.1%. Larval controls received 0.1% DMSO. T3 was used to treat embryos and larvae based on findings that T3 accumulates more effectively in embryonic zebrafish eyes than thyroxine (T4) (Tiefenbach et al., 2010). Embryos were manually dechorionated using forceps before treatments, which began at 48 h post-fertilization (hpf). Solutions were refreshed daily for treatments exceeding 24 h. To induce athyroid conditions, 2.5-day post-fertilization (dpf) *Tg(tg:nVenus-2a-nfnB)* ^*wp.rt8*^ embryos were treated with 10 mM metronidazole (MTZ) in 0.1% DMSO or 0.1% DMSO alone as a control for 24–48 h (Mackin et al., 2019). Successful athyroidy (absence of thyroid gland) was confirmed by the absence of Venus reporter-expressing thyroglobulin cells following MTZ treatment. In this study, we refer to athyroid Trβ2 gain-of-function (GOF) as having “unliganded TRβ2,” whereas co-application of Trβ2 overexpression with exogenous thyroid hormone (T3) is described as “liganded Trβ2”. This framework highlights how Trβ2 can differentially regulate opsin expression depending on ligand availability.

### 2.4 Hybridization chain reaction (HCR) *in situ* hybridization

HCR v3.0 procedures were performed following the manufacturer’s protocol (Molecular Instruments (Choi et al., 2018)). Whole larvae were fixed in 4% paraformaldehyde in phosphate-buffered saline (PBS) at 4 °C, then dehydrated and stored overnight in methanol (MeOH). Before transcript detection, tissues were rehydrated through graded MeOH/PBS/0.1% Tween washes and post-fixed in 4% paraformaldehyde in PBS. For hybridization, tissues were incubated in a hybridization oven overnight at 37 °C in a probe solution containing custom-designed, transcript-specific probes from Molecular Instruments (Supplementary Table S1). Following hybridization, excess probes were removed using the manufacturer-supplied wash buffers. Tissues were then incubated overnight at room temperature in an amplifier solution to allow chain reactions to proceed.

### 2.5 RNA extraction and quantitative RT-PCR (qPCR)

Total RNA was extracted from groups of three whole larvae or for each experimental condition using the Macherey-Nagel extraction kit. cDNA was synthesized from the isolated RNA using the Superscript IV kit with random primers (Invitrogen). Primer pairs specific to each target gene are detailed in (Supplementary Table S2). Transcript levels were quantified on an Applied Biosystems 7900 HT Fast Real-Time PCR System using SYBR-Green PCR Master Mix. Gene expression levels were compared between control and experimental groups using the ddCT method, with normalization to *β-actin* as the reference gene, following guidelines from the Applied Biosystems manual for Relative Quantitation of Gene Expression. Data analysis and visualization were performed using GraphPad Prism version 10.3.1. Statistical significance was determined using the Kruskal-Wallis Test, followed by Dunn’s multiple comparison test.

### 2.6 Antibodies and immunohistochemistry

Larval zebrafish (4- and 6-dpf) were fixed for tissue sectioning following established protocols (Barthel and Raymond, 1990). Euthanized by tricaine immersion, larvae were then fixed in 4% paraformaldehyde overnight at 4 °C. Post-fixation, larvae were washed in graded sucrose solutions and cryoprotected in 20% sucrose in phosphate-buffered saline (PBS) overnight. The larvae were then embedded in a 2:1 mixture of 20% sucrose and OCT medium (Sakura Finetek) and sectioned at 10 μm thickness on a Leica CM3050 cryostat, dehydrated and stored at −20 °C. Cryosections were thawed at room temperature for ∼10 min, blocked in antibody dilution buffer (200–250 µL per slide) for 30–60 min, and incubated overnight at 4 °C with primary antibodies. These included anti-Rhodopsin [1D1, 1:20, from Jim Fadool (Hyatt et al., 1996)], mouse monoclonal ZPR-1 for double cones, targeting Arrestin3a (Renninger et al., 2011) (1:200; Zebrafish International Resource Center/ZIRC), chicken anti-GFP (1:1,000; Abcam), anti-Red opsin (Yin et al., 2012) (1D4, 1:100–1:1,000; Abcam), and mouse anti-PKCα (A-3) for on-bipolar neurons (1:500; Santa Cruz Biotechnology) (Suzuki et al., 1990). The next day, slides were washed in PBST (PBS with 0.5% Triton-X-100) for 30 min and incubated with Cy3-or Alexa-Fluor 647-conjugated secondary antibodies (1:200; Jackson ImmunoResearch) for 1 h at RT or overnight at 4 °C. DAPI (1:1,000) was included in this step for selected experiments. After final washes in PBST and PBS, Vectashield was applied to coverslips, and slides were dried for ∼2 h before imaging, then stored at room temperature or 4 °C.

### 2.7 Confocal microscopy

Whole eyes were enucleated from larval specimens after HCR treatment and micro dissected to remove the sclera. To process tissue for sectioning, euthanized larvae were fixed overnight at 4 °C in 4% paraformaldehyde with 5% sucrose, cryoprotected in 20% sucrose, embedded in a sucrose-OCT mix, frozen, and sliced into 10 μm sections using a Leica CM4050 cryostat. The eyes were mounted in glycerol and imaged using a 20X dry- or 40X oil immersion lens on either a Nikon-Andor or Nikon-Crest spinning disk confocal microscope, both equipped with a BSI Express 16-bit sCMOS camera. A Z-series was captured from the back and front of the eye with step sizes of 0.9–3 µm to cover the entire eye globe, using Nikon Elements software. The resulting Z-stacks were flattened via maximum projection, and brightness and contrast were adjusted using FIJI (ImageJ).

### 2.8 Quantification of cells and statistical analysis

Each larval eye was labeled for at least three distinct opsin mRNA transcripts using Hybridization Chain Reaction (HCR). Confocal images of 4-day post-fertilization (dpf) larval eyes were analyzed with FIJI software. Full Z-stacks were separated into individual channels to isolate specific cone subsets and rods. Opsin mRNA + cells were counted within a 3,500 μm^2^ region of the central retina, positioned dorsally to the optic nerve head. Two to three distinct regions were sampled per retina, and the results averaged. The mean number of cones or rods (with positive signal for a specific opsin mRNA subtype), along with standard deviation (SD), was reported for at least five eyes per group. Statistical comparisons between WT (DMSO) vs. transgenic (DMSO) and WT (T3) vs. transgenic (T3) were performed using either a Student’s t-test or a Mann-Whitney U test (for data not having a normal distribution). For non-LWS cones co-labeled with *lws2*, the percentage of cells expressing both *lws2* and another specific opsin mRNA subtype was counted within the 3,500 μm^2^ region.

## 3 Results

### 3.1 Quantification of photoreceptor populations in *trβ2* gain-of-function transgenics together with thyroid hormone (TH) treatment

**FIGURE 1:**
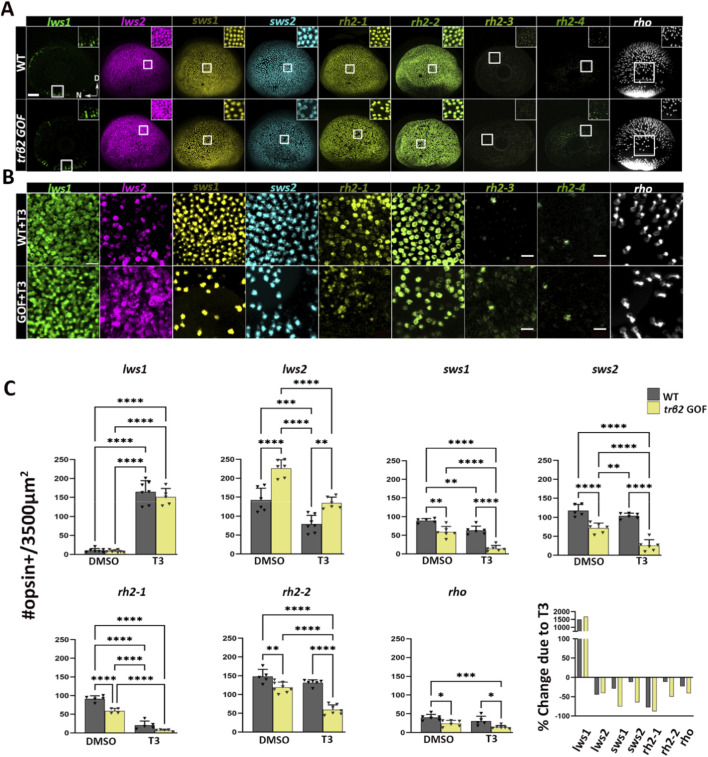
Expression patterns of nine opsin subtypes in 4dpf larval zebrafish eyes in wildtype vs. *trβ2* gain-of-function under euthyroid and hyperthyroid conditions, detected using multiplex fluorescence *in situ* hybridization chain reaction (HCR). **(A)** Representative confocal projections of whole-mounted eyes from wildtype and Tg (*crx:MYFP-2a-trβ2*) larvae. In transgenics, *lws2*+ cone density is increased at the expense of other cone opsin + subtypes and rod opsin + cells. Insets show magnified views of boxed areas to highlight the cone mosaic structure. **(B)** Opsin mRNA localization within a 3500 μm^2^ area dorsal to the optic nerve head following treatment with 100 nM T3. In transgenic, T3-treated larvae, *lws2, sws1, sws2, rh2-1,* and *rh2-2* positive cells (bottom row) are substantially reduced compared to T3-treated wildtype larvae (top row). **(C)** Scatter plot showing the average number of rod opsin+ and cone opsin + cells per unit area, quantified from confocal images of wildtype (gray) and transgenic (yellow) retinas. The final plot in C shows the % change in expression of indicated photoreceptor opsin in T3-treated vs. control, for 3,500 μm^2^ areas averaged over all samples. P-values were calculated using One-way ANOVA across four groups (WT + DMSO, WT + T3, GOF + DMSO, GOF + T3), followed by six pairwise *post hoc* comparisons (Tukey’s test). Statistical significance is represented as *P < 0.05, **P < 0.01, ***P < 0.001, ***P < 0.0001. N ≥ 6. D = Dorsal and N = Nasal. Scale bar in A = 50 μm.

We analyzed the distribution of rods and cones expressing specific opsin mRNA subtypes in wildtype (WT) and *trβ2* gain-of-function (GOF) transgenic *Tg(crx-YFP-2A*-*trβ2)* retinas (Nelson et al., 2022) at 4 dpf using fluorescence *in situ* hybridization. We further investigated potentially synergistic effects of the *crx:trβ2* transgene and exogenous thyroid hormone (TH) treatment on the photoreceptor population at this developmental stage. TH treatment was 2-4 dpf and the embryonic-larval timeframe was selected to match/for comparison with our prior work (Mitchell et al., 2015; Mackin et al., 2019; Farre et al., 2023a). Quantitative analysis, performed in a 3,500 μm^2^ region dorsal to the optic nerve head (Figures 1A–C), revealed no significant differences in numbers of *lws1*+ PRs in transgenics vs. WTs, although TH treatment greatly increased these numbers in both genetic conditions. Numbers of *lws2*+ PRs were significantly increased in transgenics vs. WTs, but all other opsin-expressing PRs (*sws1, sws2, rh2-1, rh2-2*, or *rho*), were significantly reduced in transgenics vs. WTs. TH treatment of transgenics appeared to augment several of these changes, suggesting that an interaction between TH and excess Trβ2 receptor may be important for regulation of several types of opsin genes. The *lws1/lws2* common probe (pan-*lws*) revealed no apparent differences in numbers of *lws*-expressing PRs in DMSO vs TH-treated conditions (Supplementary Figure S1B). Similarly, *lws* (pan-*lws*) transcript levels assessed by qPCR in WT groups were unchanged (Supplementary Figure S1C). By contrast, *trβ2* GOF transgenics exhibited increased pan-*lws* + photoreceptor numbers (Supplementary Figure S1B), whereas T3-treated GOF groups showed a modest reduction in comparison to GOF.

### 3.2 Co-expression of *lws2* with other cone opsin transcripts in *trβ2* gain-of-function transgenics with endogenous or supplemental TH

**FIGURE 2:**
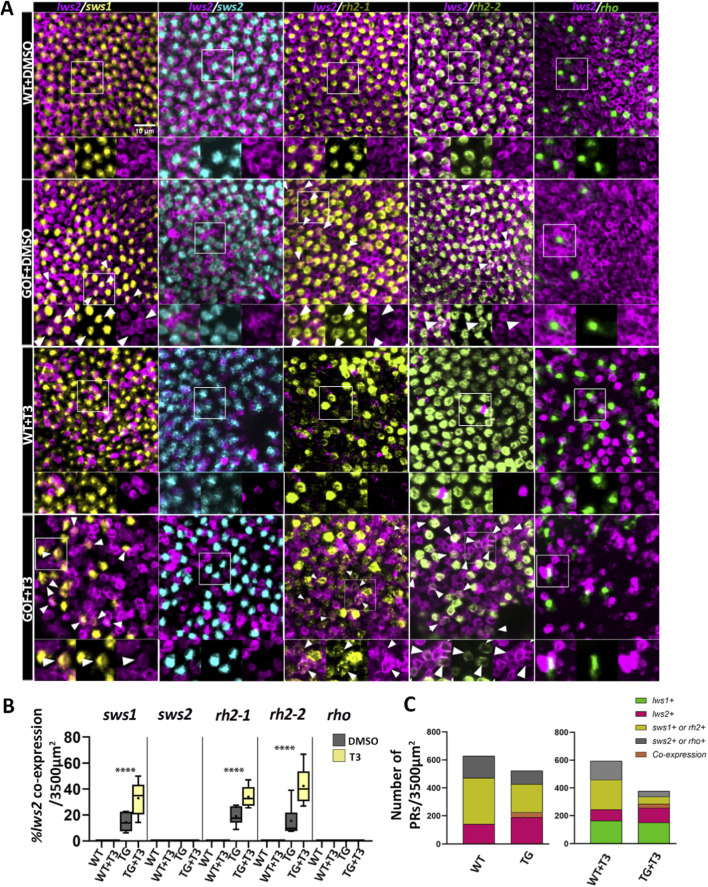
*Trβ2* gain-of-function induces co-expression of *lws2* in non-LWS cones. The proportion of photoreceptors co-labeled *lws2* and a different opsin type per unit area also increases after thyroid hormone (T3) treatment. **(A)** Fluorescence *in situ* hybridization (HCR) of 4dpf fish showing *lws2* co-expression with selected opsin transcripts (*sws1*, *sws2, rh2-1, rh2-2,* and *rho*). In wildtype fish, no co-expression with other opsin subtypes within individual cones was observed (WT + DMSO and WT + T3 rows). However, *lws2* co-expression was observed in both transgenic groups with increased proportions in the transgenic group that received T3 treatment (GOF + T3). No clear co-labeling of *sws2* (cyan) or *rho* (green) with *lws2* was observed in either transgenic group. The arrowheads indicate cells that are co-labeled for both *lws2* and another opsin subtype. Insets display enlarged regions with separate channels to highlight co-expression. **(B)** Box and whisker plot showing the percentage of cells that are positive for both *lws2* and *sws1* or *rh2-1, rh2-2, rho* per 3500 μm^2^ area dorsal to the optic nerve head. Statistical significance is represented as ****P < 0.0001. P-values were calculated using beta regression to compare co-expression proportions in transgenic DMSO and T3 groups. **(C)** Stacked bar graph of photoreceptor counts in WT and *trβ2* GOF after DMSO/T3 treatment. Colors indicate opsin expression: *lws1+* (green), *lws2+* (magenta), *sws1+* or *rh2+* (yellow), *sws2+* or *rho+* (gray), and *lws2/sws1+ or lws2/rh2+* co-expression (orange crosshatch). Scale bar = 10 μm.

To examine whether apparent observed reductions in non-LWS cones and rods were related to a transfating process, we analyzed co-expression of *lws2* with other PR opsin transcript subtypes, reasoning that transfating may result in a period of time when two distinct opsins are co-expressed within individual photoreceptors (Cheng et al., 2009; Mitchell et al., 2015; Mackin et al., 2019). Co-labeling of *lws2* with non-*lws* opsin transcripts was rare to undetectable in WT but was observed in 10%–20% of *sws1, rh2-1,* and *rh2-2*-positive cones in transgenics (Figures 2A,B), consistent with possible transfating of SWS1, Rh2-1, and Rh2-2 cones to an LWS cone phenotype. Overall, *lws +* cones accounted for ∼22% of PRs in WT and ∼43% in *trβ2* GOF transgenics, with the expansion of *lws +* cones occurring alongside proportional decreases in *rh2* and *sws1+* cones (Figure 2C).

Interestingly, no *sws2+* cones or *rho* + rods showed *lws2* co-labeling, despite significant reductions in numbers of PRs expressing *sws2* or *rho* in the transgenics. TH supplementation in WT is known to result in co-expression of *lws1* with *lws2* (Mackin et al., 2019; Farre et al., 2023b), but in the present study no other co-expression of *lws2* with any other opsin transcript was observed in TH-treated WT (Figures 2A,B). In contrast, TH supplementation in transgenics increased *lws2* co-labeling to 30%–40%, consistent with enhancing or accelerating a possible transfating process, while *sws2* and *rho*-positive PRs again rarely showed *lws2* co-expression (Figures 2A,B). Overall, *lws +* cones comprised ∼41% of PRs in WT and ∼75% in *trβ2* GOF after T3 treatment. The apparent differences in *lws +* proportions between DMSO- and T3-treated groups partly reflect double counts arising from *lws1* and *lws2* co-expression. The reduction in *sws2*+ and *rho* + PRs in transgenics and in TH-treated transgenics may be related to cell death (addressed later in Results) and/or reduced generation or differentiation of these PR types when additional *trβ2* is expressed in PRs.

Given the expansion of *lws1* expression due to TH supplementation, we examined non-*lws* cone opsin transcripts in the central retina of *trβ2* GOF transgenics for potential *lws1* co-expression. Co-labeling of *lws1* with other cone opsin transcripts was not observed in either WT or transgenics, though occasional overlap with *rh2-1* or *rh2-2* (which was challenging to definitively resolve) was noted in transgenics (Supplementary Figure S2). This finding suggests that the possible transfating due to the presence of ectopic *trβ2* promotes *lws2* but not *lws1*. Additionally, we investigated the effect of extended TH supplementation on rods since no *rho* co-labeling was detected at 4 dpf (Figure 2A). Rhodopsin and *lws* opsin (*lws1* or *lws2*) showed no clear co-localization in either WT or transgenic groups following 100 nM T3 or vehicle for 4 days, 2-6 dpf, and a more marked reduction in *rho*-expressing PRs (Supplementary Figure S3A), again consistent with either rod cell death or reduced generation/differentiation of rods. We verified that in WT, with endogenous or supplemental TH, the *sws1, rh2-1, rh2-2*, and *rho*-expressing PRs do not co-express *trβ2*, but that transgenic larval retinas display widespread expression of *trβ2* in each of these PR subtypes (Supplementary Figure S3B).

### 3.3 Effects of *crx:trβ2* and TH on retinal cell-specific proteins and cone morphology

**FIGURE 3:**
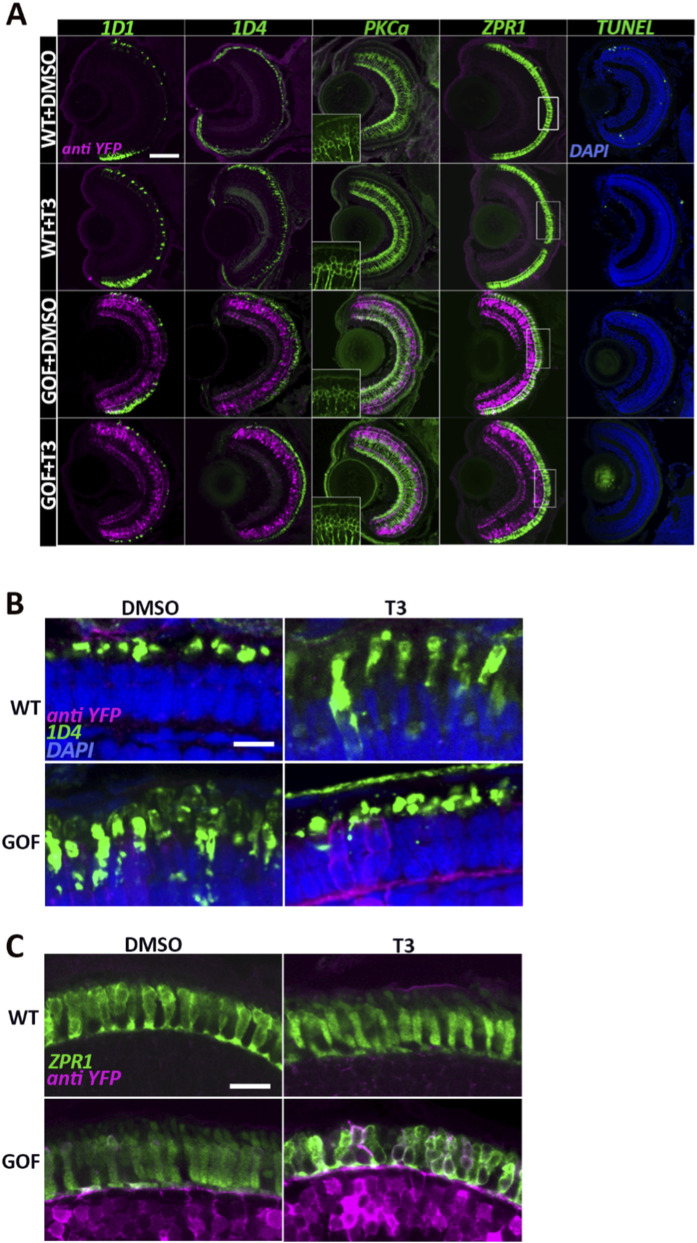
Trβ2 overexpression and T3 treatment alter cone morphology and/or distribution of selected cone antigens. **(A)** Confocal images of cryosectioned eyes from 6dpf wildtype and *trβ2* GOF transgenic fish, immunolabeled for rod opsin (*rho*, 1D1), red opsin (1D4), bipolar cells (PKCα), and double cones (ZPR1). Insets in A show the PKCα channel only. Rectangular boxes in the ZPR1 column highlight regions magnified in C. **(B)** Higher magnification of images displaying 1D4 immunolabel, showing further changes in localization of Lws opsin(s). **(C)** Higher magnification of ZPR1 immunolabel in T3-treated *trβ2* GOF transgenics, revealing changes in cone morphology and/or distribution of antigen. Scale bars in A = 50 μm, B and C = 5 μm.

Rhodopsin immunostaining (1D1 antibody) indicated reduced rod representation in transgenics, especially with T3 treatment, while red cone immunoreactivity (1D4 antibody) appeared comparatively enriched in transgenic groups, consistent with additional PRs expressing Lws opsin (Nelson et al., 2022) (Figure 3A). In the T3-treated and *trβ2* GOF samples, red cone opsin immunoreactivity extended beyond the outer segments into the inner segments, suggesting structural changes and/or changes in antigen localization associated with T3 treatment or *trβ2* overexpression (Figures 3A,B). Interestingly, this mislocalization appeared less evident in *trβ2*-overexpressing larvae also treated with T3 (Figures 3A,B).

No non-photoreceptor retinal cell types expressed Lws opsin, including the bipolar neurons that express the *crx:trβ2* transgene (Nelson et al., 2022). To further assess the effect of the *crx:trβ2* transgene on bipolar neurons, we qualitatively examined their morphology and expression of the bipolar marker PKCα protein at 6 dpf, using an anti-PKCα antibody. Bipolar neuron morphology and distribution appeared unchanged across conditions, with no detectable differences in overall organization or structure indicating that the *crx:trβ2* transgene alone is insufficient to alter the phenotypic appearance of this cell population (Figure 3A).

Given the unusual features of the Lws opsin staining pattern, the colabeling suggestive of transfating (Figure 2), and a prior report of increased width of YFP + photoreceptors of the larval *crx:trβ2-YFP* transgenic zebrafish (Nelson et al., 2022), we investigated cone morphologies and an additional red cone-selective marker by staining cryosections with the zpr1 antibody (detects Arr3a present in all LWS and Rh2 cones). Zpr1+ cones appeared to be more tightly packed, essentially filling the outer nuclear layer of GOF transgenics, while apparent gaps in the labeling pattern were evident in T3-treated WT and T3-treated GOF transgenics (Figure 3A). Higher magnification views showed normal morphologies and distributions of zpr1+ cones in WT, and slightly disrupted/more apparently cylindrical morphologies and higher densities of zpr1+ cones in GOF transgenics (Figure 3C). T3 supplementation altered morphologies in both genetic conditions, and in the *trβ2* GOF, the zpr1 antigen distribution within cones was disrupted (Figure 3C). These findings are consistent with a transfating interpretation of the opsin mRNA colabeling (Figure 2), such that nearly all cones now appear to express the zpr1 antigen.

Despite the observed changes in cone density and morphology, no differences in appearance of TUNEL-positive cells (apoptotic/dying cells), were detected across experimental groups and controls (Figure 3A). This suggests that the alterations in cone morphology and antigen expression, and the reduction in numbers of *sws2+* and *rho +* PRs were likely not due to increased cell death. Although no apoptotic cells were detected at 4 or 6 dpf, we cannot exclude transient cell death at other developmental stages.

### 3.4 Thyroid hormone (T3) mediates regulation of *lws* opsins and *trβ2* in a concentration-dependent manner

**FIGURE 4:**
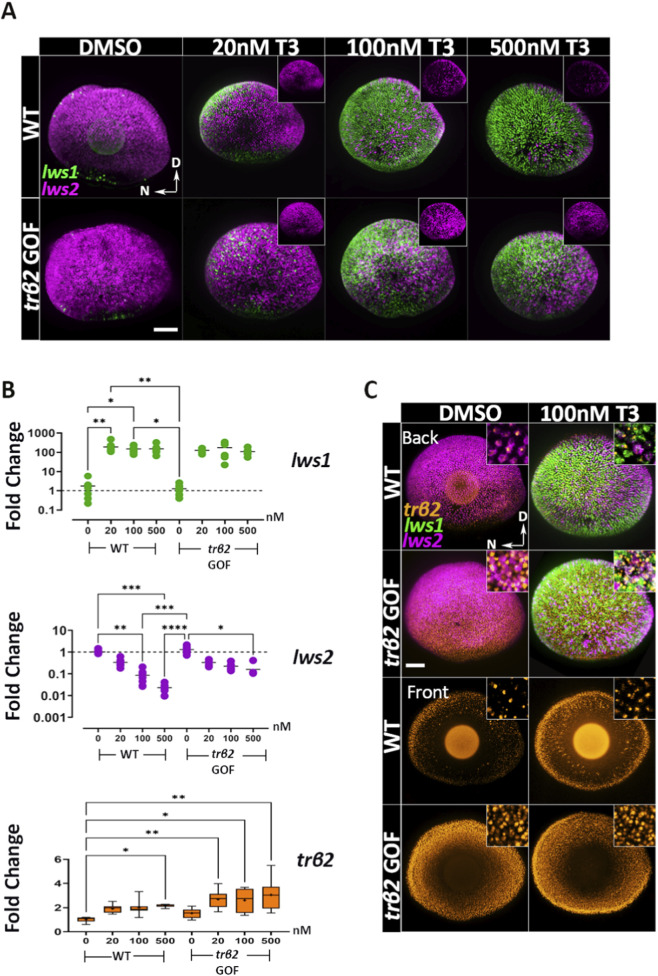
Thyroid hormone (T3) mediates concentration-dependent downregulation of *lws2* and upregulation of *trβ2* in both wildtype and *trβ2* gain-of-function transgenics. **(A)** Confocal images of whole-mount retinas from 4dpf wildtype and *trβ2* GOF transgenic larvae treated for 48 h with increasing concentrations of T3 or DMSO (control), followed by HCR *in situ* hybridization. *lws1* (green) and *lws2* (magenta) mRNA expressions are shown. N = 6 larvae. In wildtype retinas (top row), *lws2* mRNA levels progressively decrease with increasing T3 concentration. In the transgenic line (bottom row), this reduction is more subtle. Insets display *lws2* expression only. **(B)** RT-qPCR analysis of wildtype and transgenic larvae showing differential regulation of *lws1* and *lws2* following 48 h of T3 treatment. **(C)** Whole-mount retinas from wildtype and transgenic larvae showing the pattern of *trβ2* mRNA expression relative to *lws +* cones under DMSO and T3 treatment. Insets provide higher magnification of *lws* and *trβ2* co-expression. T3 upregulates *trβ2* expression in a concentration-dependent manner, while *lws2* downregulation is less pronounced in transgenic larvae compared to wildtype. Scatter and box-and-whisker plots represent fold change in transcript expression (2^-ddCT^). Each point represents a biological replicate (n ≥ 6). P-values were calculated using Kruskal-Wallis one-way ANOVA with *post hoc* testing adjusted by Dunn’s correction. Statistical significance is denoted as *P < 0.05, **P < 0.01, ***P < 0.001, ****P < 0.0001. hpt: hours post treatment. D = Dorsal N = Nasal. Scale bars in A and B = 50 μm.

Next, we investigated the expression of *lws1, lws2,* and *trβ2* as a function of exogenous T3 concentrations. We posited that the unliganded receptor (or receptor in the presence of less ligand) favors *lws2* expression, while the liganded receptor (or receptor in the presence of more ligand) promotes *lws1*. At 4 dpf, transgenic fish with normal TH levels showed increased expression of *lws2*, but not *lws1*, in comparison to WT (Figure 4A) —potentially due to a higher proportion of unliganded or partially liganded Trβ2 as TH receptors have multiple T3/T4 binding sites (Souza et al., 2014). To further test this hypothesis, we treated WT and transgenic fish with increasing concentrations of T3 (4, 20, 100, and 500 nM) for 48 h.

*Lws1* expression was significantly upregulated with the lowest concentration (4 nM T3) tested (Supplementary Figure S4A) in both WT and transgenic samples and appeared to reach a maximum in both genetic conditions at 100 nM, with 500 nM T3-treated larvae not displaying far greater expansion of the *lws1*+ domain than 100 nM T3-treated larvae (Figure 4A). Of interest, *lws2* expression showed a slight but non-significant increase at 4 nM T3 in transgenics (Supplementary Figure S4A) but progressively decreased with higher T3 concentrations in both WT and transgenic samples (Figure 4A). WT fish exhibited a more pronounced downregulation of *lws2* with increasing T3, showing reductions of 3.2-, 15.2-, and 48-fold at 20, 100, and 500 nM T3 respectively, while *lws2* expression in the *trβ2* GOF transgenic decreased by 3.2-, 4.7-, and 7.3-fold at the same concentrations (Figure 4C). This marked difference between WT and *trβ2* GOF transgenics provides some support for the hypothesis that unliganded Trβ2, with more being present in the GOF transgenics, promotes primarily *lws2*, while liganded Trβ2 promotes *lws1*.

Alternatively, or in addition, this finding may reflect an intrinsic limitation in *lws2*-expressing non-LWS-cones’ ability (in the transgenics) to switch to *lws1* expression. Additionally, we observed that *lws1* and *lws2* expression patterns in response to exogenous T3 followed a nasal-temporal gradient, with *lws1* expanding and *lws2* reducing nasally (Figure 4A)—a spatial pattern we will refer to in subsequent figures.

Because TH has been demonstrated to positively regulate its receptor(s) in other contexts (Laslo et al., 2019; Taylor et al., 2023), we evaluated transcript abundance of *trβ2* by qPCR and by HCR fluorescence in situs (Figures 4B,C). Levels of *trβ2* were indeed increased by T3 treatment in WT larvae, but only significantly (by approximately 2-fold) by the 500 nM treatment (Figure 4B). In the GOF transgenics, T3 treatment increased *trβ2* transcripts significantly (compared to WT DMSO-treated) by approximately 3-fold at all concentrations (Figure 4B). The *trβ2* transcript was co-expressed within all *lws1*+ and *lws2*+ cones in WT and *trβ2* GOF, in both DMSO and 100 nM T3-treated conditions (Figure 4C).

### 3.5 Temporal dynamics of *lws* opsin and *trβ2* regulation following TH treatment

**FIGURE 5:**
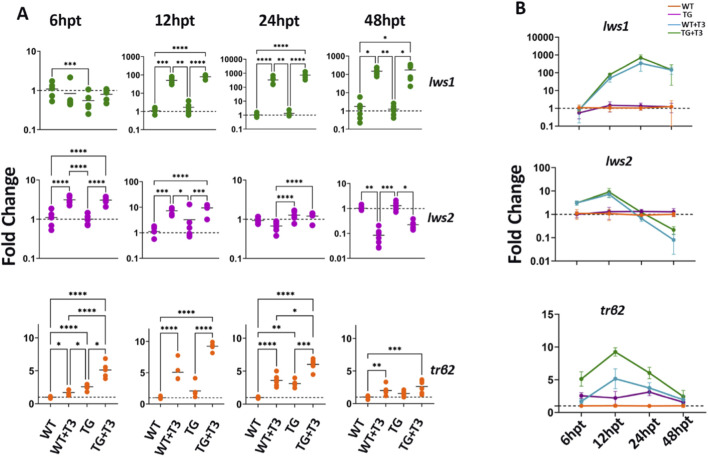
*Trβ2* upregulation precedes *lws1* activation and aligns with *lws2* downregulation). **(A)** RT-qPCR analysis of wildtype and *trβ2* GOF transgenic fish in a time-series experiment, treated with 100 nM T3, to assess the temporal dynamics of *lws1*, *lws2*, and *trβ2* mRNA expression. **(B)** Line graph showing the kinetics of fold change in *lws1, lws2*, and *trβ2* mRNA expression over time (hours post treatment, hpt). Scatter plots and line graphs represent fold changes in transcript expression (2^-ddCT). Each point in the scatter plot corresponds to a biological replicate (n ≥ 6). P-values were determined by comparing ddCT values across groups using Kruskal–Wallis one-way ANOVA with *post hoc* Dunn’s correction. Statistical significance is indicated as follows: *P < 0.05, **P < 0.01, ***P < 0.001, ****P < 0.0001. WT = wildtype; TG = transgenic overexpressing *trβ2*.

To explore the temporal relationship between TH-mediated *trβ2* regulation and *lws1/lws2* expression, we treated WT and *trβ2* GOF transgenic fish with 100 nM T3 at 2 dpf and analyzed gene expression using qPCR at select hours post-treatment (hpt). At 6 hpt, *trβ2* expression increased 1.7-fold in WT and 5-fold in transgenics, with *lws2* also showing a 3-fold increase in both groups, while *lws1* remained unchanged, indicating a selective early transcriptional response to TH (Figure 5A).

By 12 hpt, all transcripts were significantly upregulated. *Lws1* increased 50-fold in WT and 80-fold in transgenics, while *lws2* and *trβ2* showed similar increases (7–9 fold). These findings suggest TH-mediated activation of *lws1* and *lws2*, potentially via Trβ2. At 24 hpt, *lws1* reached its peak, with a 333-fold increase in WT and 735-fold in transgenics, while *lws2* stabilized, showing no significant difference from controls, particularly in WT treated with T3. These results suggest sustained *lws1* upregulation and a more transient response for *lws2*.

At 48 hpt, *lws1* levels remained elevated, with a 150-fold increase in WT and 175-fold in transgenics, but *trβ2* expression declined slightly, with a 2-fold increase in WT and 3-fold in transgenics. Interestingly, *lws2* was downregulated, showing a 15-fold reduction in WT and a 4.7-fold reduction in transgenics, which coincided with decreased *trβ2* expression. Line graphs summarized that *trβ2* upregulation peaked at 12 hpt, followed by *lws1* at 24 hpt, while *lws2* showed a transient increase before declining at 48 hpt (Figure 5B). These findings suggest Trβ2’s role as a transcriptional regulator of the *lws* locus and indicate distinct regulatory mechanisms for *lws1* and *lws2* in response to TH.

### 3.6 Trβ2 ligand-dependency in the regulation of *lws1*

**FIGURE 6:**
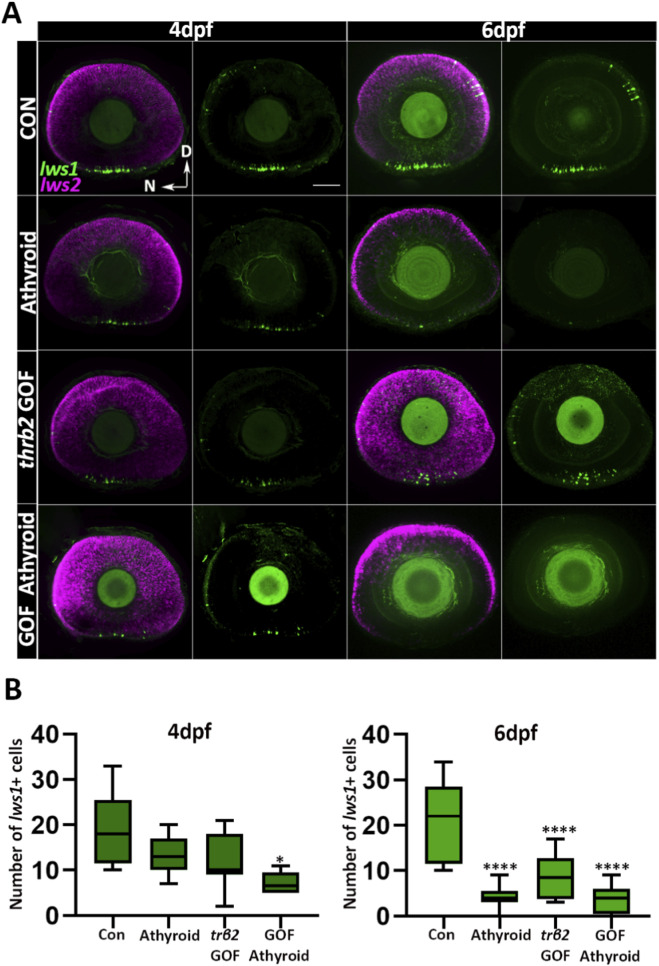
Unliganded Trβ2 delays the onset of *lws1* expression. **(A)** Confocal microscopy images of whole-mounted larval retinas from a cross between Tg (*crx: mYFP-2a-trβ2*) and homozygous Tg (*tg:nVenus-2a-nfnB*) ^wp. rt8^ transgenic lines. Larvae were treated with metronidazole or DMSO (control) for 24 h, then sorted for thyroid gland ablation at 4 or 6 dpf. **(B)** Quantification of *lws1+* cells from confocal images at 4 and 6 dpf. N = 6 larvae. P-values were calculated by comparing *lws1+* cell counts between groups using one-way ANOVA followed by Tukey’s *post hoc* test. Statistical significance is indicated as follows: *P < 0.05, **P < 0.01, ***P < 0.001, ****P < 0.0001. dpf: days post fertilization. D = Dorsal N = Nasal Scale bar = 50 μm.

Crossing *trβ2* GOF fish with *Tg(tg:nVenus-2a-nfnB)* ^*wp.rt8*^ allowed attenuation of endogenous TH levels by metronidazole treatment (McMenamin et al., 2014). At 4 dpf (treatment initiated at 2.5 dpf), no significant differences in *lws1+* cone numbers were observed between larvae with intact thyroids [normal *trβ2* levels (control)], athyroid fish lacking the *crx:trβ2* transgene (athyroid), and *trβ2* GOF fish with intact thyroids (*trβ2* GOF) (Figures 6A,B). However, athyroid *trβ2* GOF fish showed a significant reduction in *lws1*+ cone numbers compared to their counterparts with intact thyroids (Figures 6A,B). By 6 dpf (treatment from 2.5 to 6 dpf), numbers of *lws1+* cones were reduced across all groups compared to the WT controls. Athyroid groups, regardless of *trβ2* transgene presence, exhibited substantially lower *lws1+* cone numbers than *trβ2* GOF larvae with intact thyroids (Figures 6A,B). In a separate set of experiments, in which fish were treated with metronidazole or DMSO from 2.5 to 4 dpf and then sampled at 6dpf, qPCR analysis corroborated these findings. *Lws1* transcript levels mirrored the *lws1+* cone quantification trends, except for the *trβ2* GOF group, where *lws1* expression was not significantly different from the control (Supplementary Figure S4B). Interestingly, the qPCR analysis showed no significant differences in *lws2* or *trβ2* transcript levels across treatment groups compared to WT controls (Supplementary Figure S4B). These findings suggest that while liganded Trβ2 promotes *lws1* expression, unliganded Trβ2 may act through a ligand-independent manner, or through an alternative ligand (or residual TH maternally loaded into yolk) to promote *lws2* specification.

### 3.7 An intact *trβ* gene is required for T3-mediated upregulation of *lws1* and downregulation of *lws2*

To test whether T3 treatment alone, or acting through thyroid hormone receptors other than Trβ or its isoforms, could increase *lws1* expression, we analyzed a *trβ* knockout line in which LWS cones fail to fully differentiate (Volkov et al., 2020). Our findings indicate that in the absence of a functional *trβ* gene T3 treatment cannot increase *lws1* expression or repress *lws2*, as transcript levels did not differ between DMSO- and T3-treated samples and in many cases were largely undetectable (Supplementary Figure S5).

### 3.8 Spatial distribution of TH regulatory transcripts *mct8*, *dio2*, and *dio3b* in the retina and RPE

**FIGURE 7:**
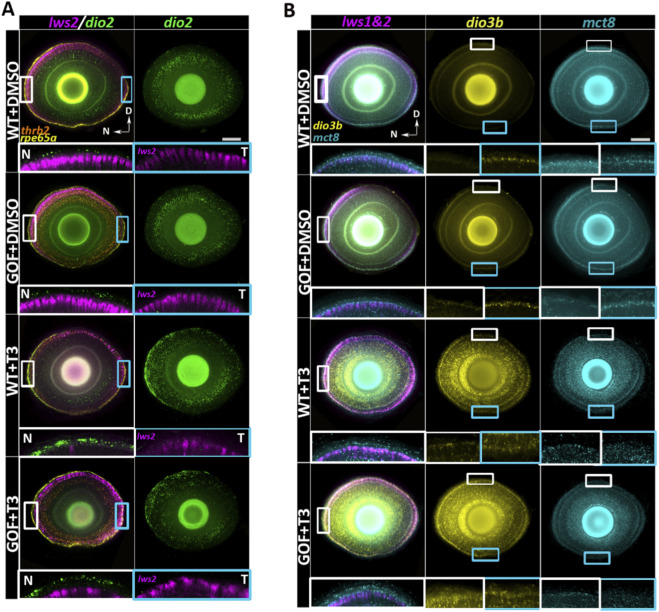
Confocal visualization of opsin and thyroid hormone-related gene expression in 4dpf wildtype and *trβ2* GOF transgenic larval retinas treated with T3 or DMSO. **(A)** Single z-projection of *lws2* expression overlaid with *dio2*, *trβ2*, and *rpe65a* labels. White rectangular boxes mark magnified areas in the N: Nasal region (left) and cyan rectangular boxes mark areas in the T: Temporal region (right), both rotated 90° (such that RPE is upper and photoreceptor layer is lower in the insets) and without *trβ2* or *rpe65a* labels. N and T panels reveal the naso-temporal patterning of *dio2* in the retinal pigment epithelium (RPE). The second column in A shows a full projection of the *dio2* channel, highlighting its naso-temporal gradient in both the RPE and neural retina, where *dio2* expression decreases from nasal to temporal regions. **(B)** Single z-projection of *lws* (labeling both *lws1* and *lws2*) expression overlaid with *dio3b* and *mct8*. Smaller white boxes within the *lws1&2* column show regions magnified and rotated 90° within the same column (such that nasal is upper, and temporal is lower in the insets). *Mct8* expression extends beyond the neural retina into the RPE, with increased expression in T3-treated groups. Larger white and cyan insets in *dio3b* and *mct8* columns magnify areas highlighted by smaller boxes, showing *dio3b* dorso-ventral patterning in the photoreceptor cell layer and *mct8* upregulation in response to T3 treatment. Scale bar = 50 μm.

Key TH-related genes (*mct8, dio2,* and *dio3b*) exhibited distinct expression patterns in the retina. At baseline, *dio2* [encoding a type-2 deiodinase that converts circulating T4 to the more biologically active T3 (Darras, 2021)] appeared enriched in the nasal/ventral retinal pigment epithelium (RPE), PRs, and inner nuclear layer (INL), following a naso-temporal gradient in both genotypes (Figure 7A). After T3 treatment of both genotypes, *dio2* expression appeared intensified in the nasal RPE and retina, accompanied by broader temporal distribution within the PR layer (Figure 7A). This finding suggests an additional positive feedback system for ocular TH signaling, such that supplemental T3 may augment the endogenous enzyme that generates T3.

Conversely, *dio3b* [ncoding a type-3 deiodinase that converts T3 into inactive catabolites (Darras, 2021)] showed minimal baseline expression in the RPE or retina, apart from weak expression in the ventral retina, in both WT and *trβ2* GOF larvae (Figure 7B). The restricted distribution of *dio3b* may reflect low endogenous TH levels at this developmental time, with the slightly elevated levels in the ventral retina potentially corresponding to a region where TH levels are higher (and promote *lws1* expression). After T3 treatment, *dio3b* expression appeared robustly induced and broadly distributed across the RPE and throughout all retinal layers, indicating strong responsiveness to altered TH levels. (Figure 7B). This response is consistent with the observed upregulation of *dio3b* in zebrafish during the 3-5 dpf period, where an increase in endogenous TH levels similarly triggers the expression of *dio3b* (Darras, 2021; Lazcano et al., 2024). TH supplementation increased and expanded expression of both *dio2* and *dio3b* in a spatially dynamic manner in both genotypes, mirroring the naso-temporal gradient observed for *lws1* expansion and *lws2* reduction. *Mct8* [encoding a TH transporter (Arjona et al., 2011; Liu et al., 2024)] was expressed in the RPE and across all retinal layers, in both WT and *trβ2* GOF larvae (Figure 7B). However, *mct8* expression levels appeared elevated within the retina following T3 treatment, indicating potential responsiveness to TH (Figure 7B).

## 4 Discussion

Thyroid hormone (TH) regulation of multichromatic color vision in vertebrates has been a topic of high interest for decades. Several TH nuclear receptors have been identified as mediators of TH-driven gene regulation within the eye (Volkov et al., 2020), with conservation across multiple species. Among these, TRβ2 plays a fundamental role in red (LWS) cone determination (Pessôa et al., 2008; Suzuki et al., 2013; Deveau et al., 2020) and may exhibit variable outcomes depending on the presence or absence of its ligand (Oberste-Berghaus et al., 2000). Here, we demonstrate that TH/Trβ2 levels increase proportions of cones co-expressing *lws2* together with other cone opsin mRNAs and differentially regulate the *lws* paralogs in zebrafish in a dose-dependent manner. Additionally, we characterize the spatial localization of key TH signaling components *dio2, dio3b*, and *mct8* in larval retina and retinal pigment epithelium (RPE).

**FIGURE 8:**
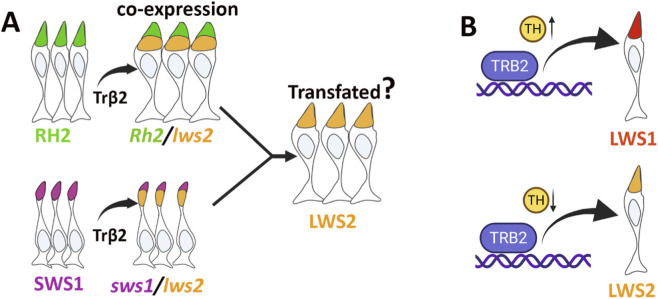
Schematic illustration of proposed Trβ2-mediated photoreceptor transfating and ligand-dependent regulation. **(A)** Trβ2 promotes *lws2* expression in RH2 and SWS1 cones, potentially leading to their transfating into LWS2 photoreceptors. **(B)** In the absence of ligand (unliganded Trβ2) or reduced ligand, *lws2* expression is predominantly enhanced. In contrast, ligand-bound Trβ2 (liganded Trβ2) preferentially upregulates *lws1* expression. Figure created with BioRender.com.

Transient ectopic expression of *trβ2* has been shown to induce red opsin expression across cone populations (Suzuki et al., 2013). However, the developmental timing of ectopic Trβ2 expression leads to variable outcomes. Trβ2 expression in differentiated cones (both native and non-native to this receptor, driven by *gnat2* promoter) resulted in an increase in red opsin immunodensity without a corresponding loss of other cone opsin types. In contrast, *crx*-driven Trβ2 expression initiated in retinal progenitor cells, led to a doubling of the red cone population at the expense of UV, blue, and green cones in zebrafish (Suzuki et al., 2013; Nelson et al., 2022). Similarly, in *Trβ* knock-in mice where *Trβ* replaced the endogenous *NRL* gene (*Nrl* ^*b2/b2*^), rod differentiation was impaired, while M-cones increased (Swaroop et al., 2010). In our study, *crx:trβ2* alone induced notable alterations in the relative numbers of photoreceptor (PR) subtypes. Increasing ligand (T3) levels upregulated *trβ2*, thereby amplifying shifts in photoreceptor subtype composition and enhancing *lws2* co-expression with other cone opsin transcripts. The intensified co-expression of *lws* opsins and the reduction in non*-lws*-expressing PRs in *trβ2* gain-of-function (GOF) transgenics suggest potential transfating of SWS1 and Rh2 type cones (Figure 8A). Consistent with this interpretation, we found widespread expression of the zpr1 antigen, Arr3a, a phototransduction component found in Rh2 and LWS cones but not in rods, SWS1 or SWS2 cones (Renninger et al., 2011). Unusual cone morphologies [noted by (Nelson et al., 2022)], exacerbated by T3 treatment, are also consistent with a transfating process, such that SWS1 cones undergoing this process may express cytoskeletal and other intracellular trafficking features more characteristic of LWS cones and experience disruptions in their maturation. An alternative to transfating is that forced ectopic expression of *trβ2* in SWS1 and Rh2 cone progenitors results in some cones maturing with a mixed fate, and others maturing with a more pronounced LWS2 fate. Since *lws* was not observed to be co-expressed with *sws2* or with *rho*, the reduction of the SWS2 and rod PR subtypes instead perhaps represents reduced generation or differentiation of additional SWS2 and rod PRs with larval growth. The only retinal cell type with forced overexpression of *trβ2* that did not show marked changes in phenotype were retinal bipolar cells, apparently unable to respond to the presence of either liganded or unliganded *trβ2* in a manner detectable by our analyses.

(Nelson et al., 2022) reported complete elimination of *sws1* cone signaling function in adult *crx:trβ2* transgenics, suggesting a full switch from *sws1* to *lws1/2* opsin expression. Likewise, blue and green cone functional responses, present in larval *crx:trβ2* transgenics, were absent in adults, where red opsin expression was exclusively amplified. We predict a similar pattern of cone function in T3-supplemented *crx:trβ2* larvae, driven by an acute increase in *trβ2* expression, comparable to the cone functional shifts observed in adult *crx:trβ2*.

Our previous findings, along with the present study, demonstrate that TH preferentially promotes *lws1* over *lws2* transcripts throughout zebrafish life history (Mackin et al., 2019; Farre et al., 2023b). As zebrafish develop/grow, endogenous TH levels increase (Heijlen et al., 2013), driving an elevated LWS1:LWS2 ratio (Mackin et al., 2019), with topographic specificity. While the nuclear receptor(s) mediating this TH-driven process remains explicitly unidentified, Trβ2 is a strong candidate, potentially evolutionarily co-opted to regulate this tandem array. Trβ2 is required for LWS cone differentiation (Suzuki et al., 2013; Deveau et al., 2020), and in the present study, is also required for upregulation of *lws1* and downregulation of *lws2* in response to T3. The traditional model of TH receptor activity suggests that ligand binding triggers coactivator recruitment, while ligand absence leads to corepressor recruitment for genes that are positively regulated by TH (Nicolini et al., 2024). However, the function of the Trβ2 receptor has been found to deviate from this canonical mechanism. There is evidence that both the liganded and unliganded forms of Trβ2 can activate the transcription of positively regulated genes, with the liganded form showing higher levels of activity (Oberste-Berghaus et al., 2000). Similarly, its splice variant Trβ1 modulates transcriptional activity by influencing levels of co-activators and co-repressors through mechanisms distinct from the canonical cofactor switching mechanism (Shabtai et al., 2021). Trβ2 in the zebrafish may represent another deviation to permit differential regulation of a tandemly-duplicated array (Figure 8B).

Temporal analysis of *lws1*, *lws2*, and *trβ*2 expression during TH treatment revealed a transcriptional succession. The early upregulation of *trβ2* and *lws2* suggests a rapid response to TH, possibly reflecting initial *lws2* activation by the increase in unliganded receptors. In contrast, later, sustained *lws1* upregulation, particularly in *trβ2* GOF transgenics, followed by suppression of *lws2*, illustrates distinct temporal phases of TH-mediated LWS cone gene regulation.

These findings underscore the dynamic nature of TH signaling in the retina, where liganded and unliganded Trβ2 differentially regulate *lws1* and *lws2* expression. The enhanced effects of TH on *lws1* expression in *trβ2* GOF transgenics may result from increased Trβ2 abundance, as indicated by elevated transcript levels. This amplification likely reflects a positive feedback mechanism, wherein TH upregulates its receptor to further enhance *lws1* expression. This mechanism is reminiscent of previous reports demonstrating TH-mediated upregulation of TRβ1 in the mouse heart (Sadow et al., 2003). The persistence of *lws2* expression in GOF transgenics, even under high TH concentrations, suggests additional regulatory constraints, such as intrinsic restricted plasticity of non-LWS cones or the saturation of *lws1* expression capacity, which may limit the complete reprogramming of these cells. The absence of any regulatory effect of T3 on *lws1* or *lws2* in *trβ* knockouts supports the hypothesis that Trβ and/or its Trβ2 isoform is required for T3-mediated control of the *lws* locus. However, a conditional knockout of *trβ2* after LWS cone differentiation will be essential to fully elucidate the distinct roles of liganded and unliganded Trβ2 in opsin regulation, because LWS cones fail to fully differentiate in the *trβ* knockout (Volkov et al., 2020). The activity of TH/Trβ2 could be in concert with multiple factors to regulate *lws1* vs *lws2* (Volkov et al., 2024).

Deiodinases and TH transporters are critical regulators of intracellular TH availability, and their expression is tightly coordinated during vertebrate development, including in zebrafish (Arjona et al., 2011; Heijlen et al., 2013), and are largely conserved across species (Darras and Van Herck, 2012). In zebrafish, the distribution of expression of key TH-related genes—*dio2*, *dio3b*, and *mct8*—are established prior to endogenous T4 production, which begins around 3 days post-fertilization (dpf) (Heijlen et al., 2013). Previous studies reported low but stable expression levels of *dio2* as early as 8hpf, with a marked upregulation observed post-hatching. In contrast, maternally deposited *dio3b* transcripts are detectable as early as 1hpf, with a notable decline by 24hpf. A subsequent sharp increase in *dio3b* expression between 4–5dpf, has also been documented. These developmental patterns are consistent with the measured fluctuations in T3 and T4 levels during the embryonic-to-larval transition in zebrafish (Walpita et al., 2007; Heijlen et al., 2013). Our results indicate that T3 supplementation appear to positively regulate both *dio2* and *dio3b* expression in the retina and RPE. T3 treatment resulted in an upregulation of *dio2*, particularly within the nasal RPE and PR layer, along with an expansion of its expression domain toward the temporal retina. This pattern suggests a positive feedback mechanism in ocular TH signaling, whereby exogenous T3 enhances expression of the enzyme that locally generates active T3. The spatial specificity of *dio2* and *dio3b* expression in the retina and RPE, however, also implies the involvement of additional regulatory factors or signaling pathways. TH-induced upregulation of *dio3b* and *mct8* were observed across all retinal layers and the RPE, indicating a robust, widespread transcriptional response to elevated T3 levels. Given that *dio3b* encodes a deiodinase that inactivates T3, its upregulation likely serves a compensatory, protective role—modulating local TH levels to prevent overactivation of TH-sensitive pathways. Similar mechanisms have been described in the mouse retina, where Dio3 protects cones from TH-induced toxicity (Ng et al., 2010). Interestingly, overexpression of *trβ2* did not appear to alter the expression of *dio2, dio3b*, or *mct8*, suggesting that transcriptional regulation of these TH regulatory genes may be mediated by other TH receptors. We also wish to acknowledge that the majority of studies presented in the current work utilize the GOF approach, which may trigger events that do not occur physiologically. A conditional LOF approach, one that allows LWS cones to initially differentiate, may address this issue in the future.

Numerous studies in zebrafish have uncovered and clarified the function of the repertoire of transcription factors critical for the generation of specific vertebrate cone subtypes. *Trβ2, foxq2, and tbx2b,* promote the differentiation of LWS, SWS2, and SWS1 cones, respectively (Alvarez-Delfin et al., 2009; Volkov et al., 2020; Ogawa et al., 2021; Neil et al., 2024), while *six6* and *six7* participate in promoting differentiation of Rh2 and SWS2 cones (Ogawa et al., 2019). Of these factors, only Trβ2 is known to interact with a ligand, making Trβ2 an attractive candidate as a mediator of changes to the color vision system that are topographically regulated (through local control of ligand availability) and that accompany other anatomical/physiological changes over developmental time (through endocrine production of ligand). The present study finds further support for such ligand-dependent functions of Trβ2.

## Data Availability

The raw data supporting the conclusions of this article will be made available by the authors, without undue reservation.

## References

[B1] Alvarez-Delfin K. Morris A. C. Snelson C. D. Gamse J. T. Gupta T. Marlow F. L. (2009). Tbx2b is required for ultraviolet photoreceptor cell specification during zebrafish retinal development. Proc. Natl. Acad. Sci. 106 (6), 2023–2028. 10.1073/pnas.0809439106 19179291 PMC2632714

[B2] Arjona F. J. de Vrieze E. Visser T. J. Flik G. Klaren P. H. (2011). Identification and functional characterization of zebrafish solute carrier Slc16a2 (Mct8) as a thyroid hormone membrane transporter. Endocrinology 152 (12), 5065–5073. 10.1210/en.2011-1166 21952246

[B3] Baden T. Osorio D. (2019). The retinal basis of vertebrate color vision. Annu. Rev. Vis. Sci. 5 (1), 177–200. 10.1146/annurev-vision-091718-014926 31226010

[B4] Barthel L. K. Raymond P. A. (1990). Improved method for obtaining 3-microns cryosections for immunocytochemistry. J. Histochem. Cytochem. 38 (9), 1383–1388. 10.1177/38.9.2201738 2201738

[B5] Bowmaker J. K. (2008). Evolution of vertebrate visual pigments. Vis. Res. 48 (20), 2022–2041. 10.1016/j.visres.2008.03.025 18590925

[B6] Cheng C. L. Gan K. J. Flamarique I. N. (2009). Thyroid hormone induces a time-dependent opsin switch in the retina of salmonid fishes. Investigative Ophthalmol. Vis. Sci. 50 (6), 3024–3032. 10.1167/iovs.08-2713 19218617

[B7] Chinen A. Hamaoka T. Yamada Y. Kawamura S. (2003). Gene duplication and spectral diversification of cone visual pigments of zebrafish. Genetics 163 (2), 663–675. 10.1093/genetics/163.2.663 12618404 PMC1462461

[B8] Choi H. M. T. Schwarzkopf M. Fornace M. E. Acharya A. Artavanis G. Stegmaier J. (2018). Third-generation *in situ* hybridization chain reaction: multiplexed, quantitative, sensitive, versatile, robust. Development 145 (12), dev165753. 10.1242/dev.165753 29945988 PMC6031405

[B9] Darras V. M. (2021). Deiodinases: how nonmammalian research helped shape our present view. Endocrinology 162 (6), bqab039. 10.1210/endocr/bqab039 33606002 PMC8143656

[B10] Darras V. M. Van Herck S. (2012). Iodothyronine deiodinase structure and function: from ascidians to humans. J. Endocrinol. 215 (2), 189–206. 10.1530/JOE-12-0204 22825922

[B11] Davies W. I. Collin S. P. Hunt D. M. (2012). Molecular ecology and adaptation of visual photopigments in craniates. Mol. Ecol. 21 (13), 3121–3158. 10.1111/j.1365-294X.2012.05617.x 22650357

[B12] Deveau C. Jiao X. Suzuki S. C. Krishnakumar A. Yoshimatsu T. Hejtmancik J. F. (2020). Thyroid hormone receptor beta mutations alter photoreceptor development and function in *Danio rerio* (zebrafish). PLoS Genet. 16 (6), e1008869. 10.1371/journal.pgen.1008869 32569302 PMC7332105

[B13] Farre A. A. Sun C. Starostik M. R. Hunter S. S. English M. A. Duncan A. (2023a). Long wavelength-sensing cones of zebrafish retina exhibit multiple layers of transcriptional heterogeneity. Front. Cell. Neurosci. 17, 1214084. 10.3389/fncel.2023.1214084 37519633 PMC10382231

[B14] Farre A. A. Thomas P. Huang J. Poulsen R. A. Owusu Poku E. Stenkamp D. L. (2023b). Plasticity of cone photoreceptors in adult zebrafish revealed by thyroid hormone exposure. Sci. Rep. 13 (1), 15697. 10.1038/s41598-023-42686-x 37735192 PMC10514274

[B15] Hadyniak S. E. Hagen J. F. Eldred K. C. Brenerman B. Hussey K. A. McCoy R. C. (2024). Retinoic acid signaling regulates spatiotemporal specification of human green and red cones. PLoS Biol. 22 (1), e3002464. 10.1371/journal.pbio.3002464 38206904 PMC10783767

[B16] Hayashi T. Motulsky A. G. Deeb S. S. (1999). Position of a'green-red'hybrid gene in the visual pigment array determines colour-vision phenotype. Nat. Genet. 22 (1), 90–93. 10.1038/8798 10319869

[B17] Heijlen M. Houbrechts A. M. Darras V. M. (2013). Zebrafish as a model to study peripheral thyroid hormone metabolism in vertebrate development. General Comp. Endocrinol. 188, 289–296. 10.1016/j.ygcen.2013.04.004 23603432

[B18] Hofmann C. M. Carleton K. L. (2009). Gene duplication and differential gene expression play an important role in the diversification of visual pigments in fish. Integr. Comp. Biol. 49 (6), 630–643. 10.1093/icb/icp079 21665846

[B19] Hunt D. M. Dulai K. S. Cowing J. A. Julliot C. Mollon J. D. Bowmaker J. K. (1998). Molecular evolution of trichromacy in Primates. Vis. Res. 38 (21), 3299–3306. 10.1016/s0042-6989(97)00443-4 9893841

[B20] Hyatt G. A. Schmitt E. A. Fadool J. M. Dowling J. E. (1996). Retinoic acid alters photoreceptor development *in vivo* . Proc. Natl. Acad. Sci. 93 (23), 13298–13303. 10.1073/pnas.93.23.13298 8917585 PMC24087

[B21] Laslo M. Denver R. J. Hanken J. (2019). Evolutionary conservation of thyroid hormone receptor and deiodinase expression dynamics in ovo in a direct-developing frog, Eleutherodactylus coqui. Front. Endocrinol. 10, 307. 10.3389/fendo.2019.00307 31178826 PMC6542950

[B22] Lazcano I. Pech-Pool S. M. Maldonado-Lira M. F. Olvera A. Darras V. M. Orozco A. (2024). Ontogeny of thyroid hormone signaling in the retina of zebrafish: effects of thyroidal status on retinal morphology, cell survival, and color preference. Int. J. Mol. Sci. 25 (22), 12215. 10.3390/ijms252212215 39596289 PMC11594673

[B23] Liu Y. Ng L. Liu H. Heuer H. Forrest D. (2024). Cone photoreceptor differentiation regulated by thyroid hormone transporter MCT8 in the retinal pigment epithelium. Proc. Natl. Acad. Sci. 121 (30), e2402560121. 10.1073/pnas.2402560121 39018199 PMC11287251

[B24] Mackin R. D. Frey R. A. Gutierrez C. Farre A. A. Kawamura S. Mitchell D. M. (2019). Endocrine regulation of multichromatic color vision. Proc. Natl. Acad. Sci. U. S. A. 116 (34), 16882–16891. 10.1073/pnas.1904783116 31383755 PMC6708328

[B25] McMenamin S. K. Bain E. J. McCann A. E. Patterson L. B. Eom D. S. Waller Z. P. (2014). Thyroid hormone-dependent adult pigment cell lineage and pattern in zebrafish. Science 345 (6202), 1358–1361. 10.1126/science.1256251 25170046 PMC4211621

[B26] Mitchell D. M. Stevens C. B. Frey R. A. Hunter S. S. Ashino R. Kawamura S. (2015). Retinoic acid signaling regulates differential expression of the tandemly-duplicated long wavelength-sensitive cone opsin genes in zebrafish. PLoS Genet. 11 (8), e1005483. 10.1371/journal.pgen.1005483 26296154 PMC4546582

[B27] Musilova Z. Cortesi F. Matschiner M. Davies W. I. Patel J. S. Stieb S. M. (2019). Vision using multiple distinct rod opsins in deep-sea fishes. Science 364 (6440), 588–592. 10.1126/science.aav4632 31073066 PMC6628886

[B28] Nathans J. Thomas D. Hogness D. S. (1986). Molecular genetics of human color vision: the genes encoding blue, green, and red pigments. Science 232 (4747), 193–202. 10.1126/science.2937147 2937147

[B29] Neil G. J. Kluttig K. H. Allison W. T. (2024). Determining photoreceptor cell identity: rod *versus* cone fate governed by tbx2b opposing nrl. Investigative Ophthalmol. Vis. Sci. 65 (1), 39. 10.1167/iovs.65.1.39 38261312 PMC10810017

[B30] Nelson R. F. Balraj A. Suresh T. Elias L. J. Yoshimatsu T. Patterson S. S. (2022). The developmental progression of eight ops n spectral signals recorded from the zebrafish retinal cone layer is altered by the timing and cell type expression of thyroxin receptor β2 (trβ2) gain-of-function transgenes. Eneuro 9 (6), 25. 10.1523/eneuro.0326-22.2022 PMC9718360 36351817

[B31] Ng L. Lyubarsky A. Nikonov S. S. Ma M. Srinivas M. Kefas B. (2010). Type 3 deiodinase, a thyroid-hormone-inactivating enzyme, controls survival and maturation of cone photoreceptors. J. Neurosci. 30 (9), 3347–3357. 10.1523/JNEUROSCI.5267-09.2010 20203194 PMC2843520

[B32] Ng L. Lu A. Swaroop A. Sharlin D. S. Swaroop A. Forrest D. (2011). Two transcription factors can direct three photoreceptor outcomes from rod precursor cells in mouse retinal development. J. Neurosci. 31 (31), 11118–11125. 10.1523/JNEUROSCI.1709-11.2011 21813673 PMC3158567

[B33] Nicolini G. Casini G. Posarelli C. Amato R. Lulli M. Balzan S. (2024). Thyroid hormone signaling in retinal development and function: implications for diabetic retinopathy and age-related macular degeneration. Int. J. Mol. Sci. 25 (13), 7364. 10.3390/ijms25137364 39000471 PMC11242054

[B34] Oberste-Berghaus C. Zanger K. Hashimoto K. Cohen R. N. Hollenberg A. N. Wondisford F. E. (2000). Thyroid hormone-independent interaction between the thyroid hormone receptor beta2 amino terminus and coactivators. J. Biol. Chem. 275 (3), 1787–1792. 10.1074/jbc.275.3.1787 10636876

[B35] Ogawa Y. Corbo J. C. (2021). Partitioning of gene expression among zebrafish photoreceptor subtypes. Sci. Rep. 11 (1), 17340. 10.1038/s41598-021-96837-z 34462505 PMC8405809

[B36] Ogawa Y. Shiraki T. Asano Y. Muto A. Kawakami K. Suzuki Y. (2019). Six6 and Six7 coordinately regulate expression of middle-wavelength opsins in zebrafish. Proc. Natl. Acad. Sci. U. S. A. 116 (10), 4651–4660. 10.1073/pnas.1812884116 30765521 PMC6410792

[B37] Ogawa Y. Shiraki T. Fukada Y. Kojima D. (2021). Foxq2 determines blue cone identity in zebrafish. Sci. Adv. 7 (41), eabi9784. 10.1126/sciadv.abi9784 34613771 PMC8494292

[B38] Peng G.-H. Chen S. (2011). Active opsin loci adopt intrachromosomal loops that depend on the photoreceptor transcription factor network. Proc. Natl. Acad. Sci. 108 (43), 17821–17826. 10.1073/pnas.1109209108 22006320 PMC3203788

[B39] Pessôa C. N. Santiago L. A. Santiago D. A. Machado D. S. Rocha F. A. Ventura D. F. (2008). Thyroid hormone action is required for normal cone opsin expression during mouse retinal development. Investigative Ophthalmol. Vis. Sci. 49 (5), 2039–2045. 10.1167/iovs.07-0908 18436838

[B40] Renninger S. L. Gesemann M. Neuhauss S. C. (2011). Cone arrestin confers cone vision of high temporal resolution in zebrafish larvae. Eur. J. Neurosci. 33 (4), 658–667. 10.1111/j.1460-9568.2010.07574.x 21299656

[B41] Roberts M. R. Hendrickson A. McGuire C. R. Reh T. A. (2005). Retinoid X receptor (gamma) is necessary to establish the S-opsin gradient in cone photoreceptors of the developing mouse retina. Invest Ophthalmol. Vis. Sci. 46 (8), 2897–2904. 10.1167/iovs.05-0093 16043864

[B42] Roberts M. R. Srinivas M. Forrest D. Morreale de Escobar G. Reh T. A. (2006). Making the gradient: thyroid hormone regulates cone opsin expression in the developing mouse retina. Proc. Natl. Acad. Sci. 103 (16), 6218–6223. 10.1073/pnas.0509981103 16606843 PMC1458858

[B43] Sadow P. M. Chassande O. Koo E. K. Gauthier K. Samarut J. Xu J. (2003). Regulation of expression of thyroid hormone receptor isoforms and coactivators in liver and heart by thyroid hormone. Mol. Cell. Endocrinol. 203 (1-2), 65–75. 10.1016/s0303-7207(03)00122-9 12782404

[B44] Shabtai Y. Nagaraj N. K. Batmanov K. Cho Y.-W. Guan Y. Jiang C. (2021). A coregulator shift, rather than the canonical switch, underlies thyroid hormone action in the liver. Genes Dev. 35 (5-6), 367–378. 10.1101/gad.345686.120 33602873 PMC7919419

[B45] Smallwood P. M. Wang Y. Nathans J. (2002). Role of a locus control region in the mutually exclusive expression of human red and green cone pigment genes. Proc. Natl. Acad. Sci. U. S. A. 99 (2), 1008–1011. 10.1073/pnas.022629799 11773636 PMC117421

[B46] Souza P. C. T. Puhl A. C. Martínez L. Aparício R. Nascimento A. S. Figueira A. C. M. (2014). Identification of a new hormone-binding site on the surface of thyroid hormone receptor. Mol. Endocrinol. 28 (4), 534–545. 10.1210/me.2013-1359 24552590 PMC5414925

[B47] Suzuki S. Tachibana M. Kaneko A. (1990). Effects of glycine and GABA on isolated bipolar cells of the mouse retina. J. Physiology 421 (1), 645–662. 10.1113/jphysiol.1990.sp017967 1693403 PMC1190107

[B48] Suzuki S. C. Bleckert A. Williams P. R. Takechi M. Kawamura S. Wong R. O. (2013). Cone photoreceptor types in zebrafish are generated by symmetric terminal divisions of dedicated precursors. Proc. Natl. Acad. Sci. U. S. A. 110 (37), 15109–15114. 10.1073/pnas.1303551110 23980162 PMC3773785

[B49] Swaroop A. Kim D. Forrest D. (2010). Transcriptional regulation of photoreceptor development and homeostasis in the mammalian retina. Nat. Rev. Neurosci. 11 (8), 563–576. 10.1038/nrn2880 20648062 PMC11346175

[B50] Takechi M. Kawamura S. (2005). Temporal and spatial changes in the expression pattern of multiple red and green subtype opsin genes during zebrafish development. J. Exp. Biol. 208 (Pt 7), 1337–1345. 10.1242/jeb.01532 15781894

[B51] Taylor E. Wynen H. Heyland A. (2023). Thyroid hormone membrane receptor binding and transcriptional regulation in the sea urchin *Strongylocentrotus purpuratus* . Front. Endocrinol. 14, 1195733. 10.3389/fendo.2023.1195733 37305042 PMC10250714

[B52] Tiefenbach J. Moll P. R. Nelson M. R. Hu C. Baev L. Kislinger T. (2010). A live zebrafish-based screening system for human nuclear receptor ligand and cofactor discovery. PloS one 5 (3), e9797. 10.1371/journal.pone.0009797 20339547 PMC2842432

[B53] Volkov L. I. Kim-Han J. S. Saunders L. M. Poria D. Hughes A. E. O. Kefalov V. J. (2020). Thyroid hormone receptors mediate two distinct mechanisms of long-wavelength vision. Proc. Natl. Acad. Sci. U. S. A. 117 (26), 15262–15269. 10.1073/pnas.1920086117 32541022 PMC7334509

[B54] Volkov L. I. Ogawa Y. Somjee R. Vedder H. E. Powell H. E. Poria D. (2024). Samd7 represses short-wavelength cone genes to preserve long-wavelength cone and rod photoreceptor identity. Proc. Natl. Acad. Sci. 121 (47), e2402121121. 10.1073/pnas.2402121121 39531499 PMC11588049

[B55] Walpita C. N. Van der Geyten S. Rurangwa E. Darras V. M. (2007). The effect of 3, 5, 3′-triiodothyronine supplementation on zebrafish (*Danio rerio*) embryonic development and expression of iodothyronine deiodinases and thyroid hormone receptors. General Comp. Endocrinol. 152 (2-3), 206–214. 10.1016/j.ygcen.2007.02.020 17418841

[B56] Wärnmark A. Treuter E. Wright A. P. Gustafsson J.-A. (2003). Activation functions 1 and 2 of nuclear receptors: molecular strategies for transcriptional activation. Mol. Endocrinol. 17 (10), 1901–1909. 10.1210/me.2002-0384 12893880

[B57] Westerfield M. (2007). The zebrafish book: A guide for the laboratory use of zebrafish (Danio Rerio). Oregon: University of Oregon Press.

[B58] Yin J. Brocher J. Linder B. Hirmer A. Sundaramurthi H. Fischer U. (2012). The 1D4 antibody labels outer segments of long double cone but not rod photoreceptors in zebrafish. Investigative Ophthalmol. Vis. Sci. 53 (8), 4943–4951. 10.1167/iovs.12-9511 22743318

