# Supplementary material for: Thyroid hormone receptor beta-2 (TRβ2) overexpression modulates photoreceptor phenotype diversity in a ligand-dependent manner
Source: Front Cell Dev Biol. 2025 Oct 20;13:1697930. doi: 10.3389/fcell.2025.1697930 (PMC12581212; doi:10.3389/fcell.2025.1697930)
Supplement: Supplementary file 1 [file DataSheet1.pdf]

Thyroid hormone receptor beta-2 (TRβ2) overexpression modulates photoreceptor phenotype diversity in a ligand-dependent manner: Supplementary Material

Supplementary Table S1: NCBI Accession Number for generating HCR probe sets and Molecular Instruments lot numbers.

Gene Name	NCBI Accession Number	Lot Number
opn1lw1	NM_001313715.1	RTG197
opn1lw2	NM_001002443.2	PRE786
opn1mw1	NM_131253.2	PRE774
opn1mw2	NM_182891.2	PRE775
opn1mw3	NM_182892.2	PRR345
opn1mw4	NM_131254.1	PRR346
opnsw1	NM_131319.2	PRO929
opnsw2	NM_131192.2	PRO930
rho	NM_131084.2	RTN120
trβ2	AB759513	RTI795
dio2	NM_212789.4	PRE784
dio3b	NM_001177935.3	PRE783
mct8	NM_001258230.1	RTJ712
rpe65a	NM_200751.1	RTN922
lws1/2		PRR343

Supplementary Table S2: Primers for qPCR.

Gene Name	Forward Primer	Reverse Primer
opn1lw1	CCCACACTGCATCTCGACAA	AAGGTATTCCCCATCACTCCAA
opn1lw2	AGAGGGAAGAACTGGACTTTCAGA	TTCAGAGGAGTTTTGCCTACATATGT
trβ2	TCGACGCACAATTCAGAAGA	CTCAATCAGCTTCCGCTTGG
beta actin	GTACCACCAGACAATACAGT	CTTCTTGGGTATGGAATCTTGC

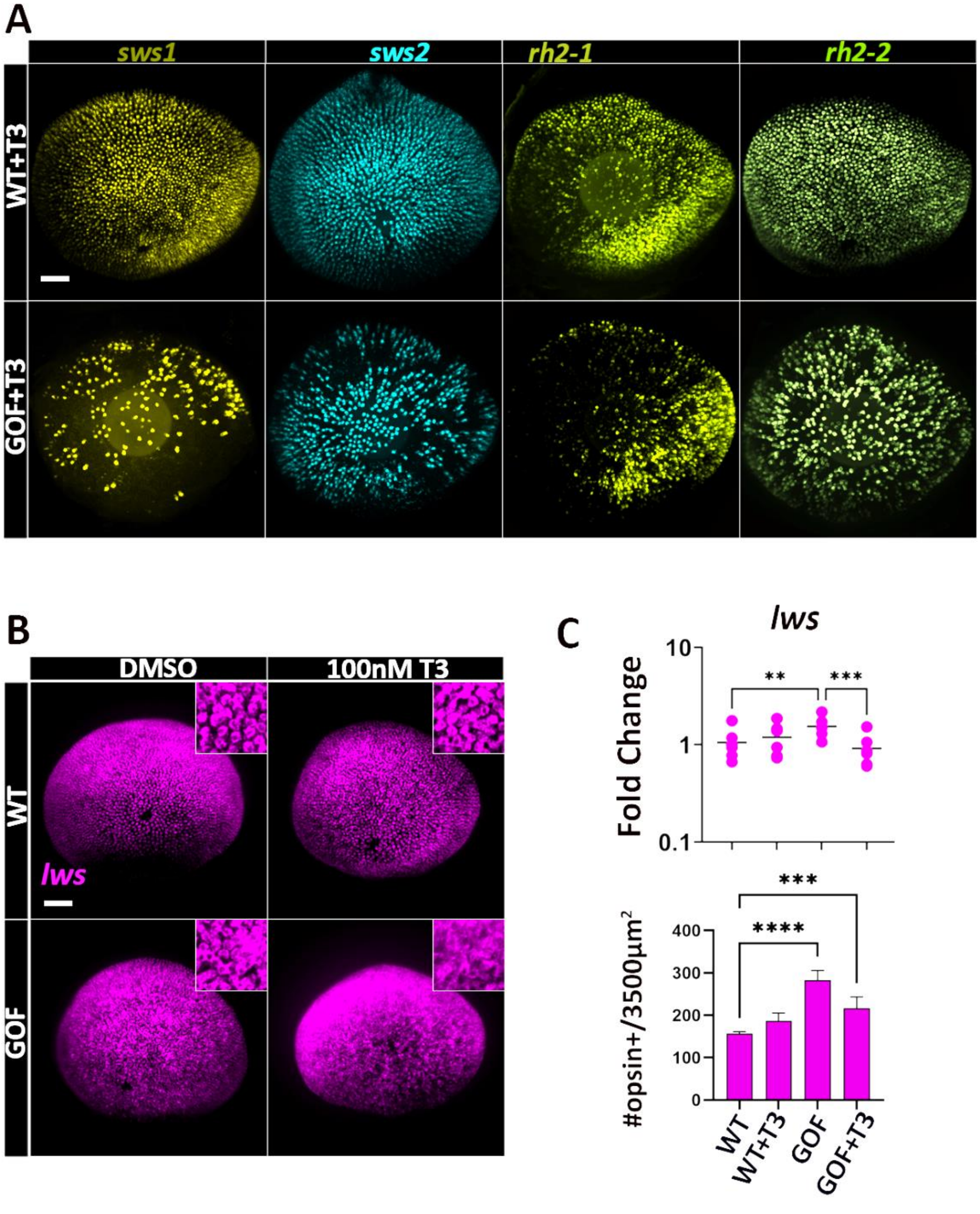

Supplemental Figure S1. Expression patterns of specific opsin subtypes in 4dpf larval zebrafish eyes following 48 hours of 100nM T3 treatment, analyzed by multiplex fluorescence in situ hybridization chain reaction (HCR) or RT-qPCR.

A) Full confocal projections of wildtype and *crx:myfp-2a-trβ2* transgenic larval eyes, showing a marked reduction in cone density in the *trβ2* GOF line compared to wildtype clutch-mates. T3 treatment induces a naso-temporal gradient in opsin transcript expression, which is more pronounced in the transgenic line. B) T3-treated transgenic larvae exhibit increased *LWS* mRNA localization using probe sets that detect both *lws1* and *lws2*. C) RT-qPCR analysis and

quantification of *lws* transcripts following T3 or DMSO treatment. Scatter plots show fold change expression (2^-ΔΔCT), with each point representing a biological replicate ($n \geq 6$). Column graph shows the average number of *lws* opsin+ cells per unit area, quantified from confocal images. P-values were calculated by comparing ddCT values between groups using Kruskal-Wallis one-way ANOVA with post-hoc Dunn's correction or ordinary One-way ANOVA with Dunnett's multiple comparisons test. Statistical significance is indicated as follows: *$P < 0.05$, **$P < 0.01$, ***$P < 0.001$, ****$P < 0.0001$. hpt: hours post treatment. Scale bar = 50μm.

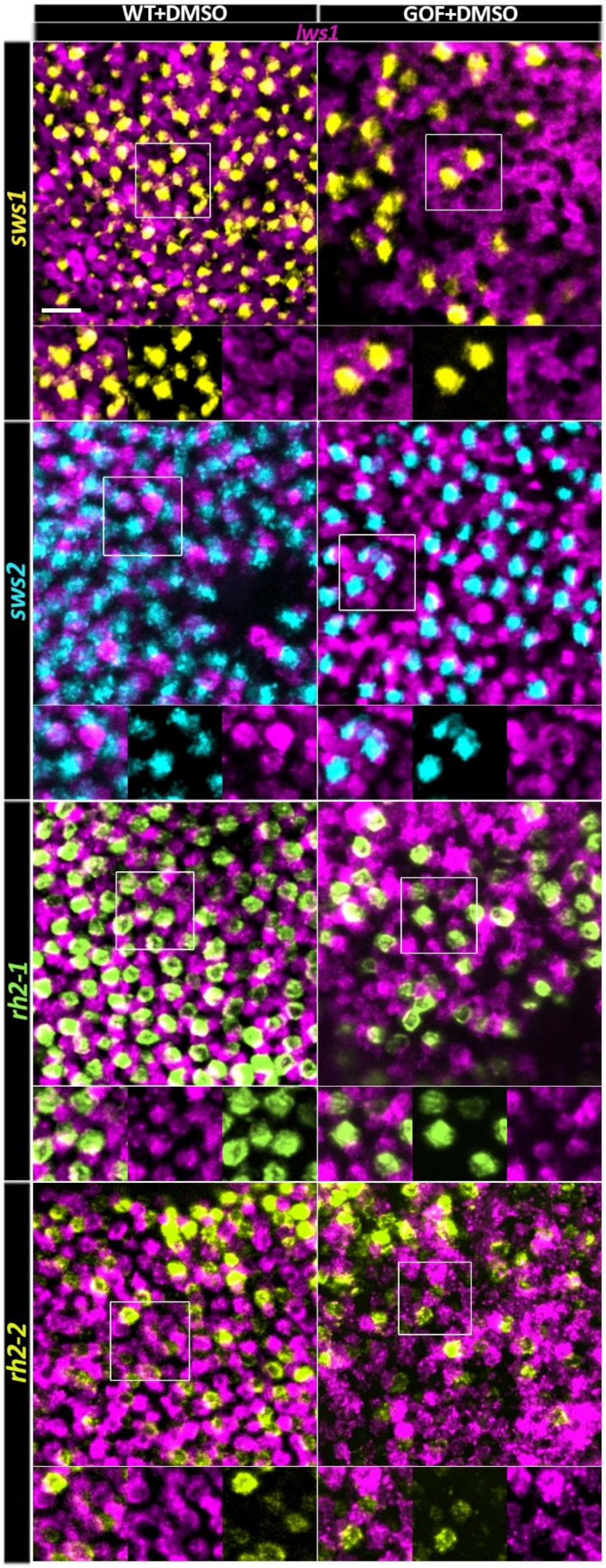

Supplemental Figure S2. Fluorescence in situ hybridization (HCR) of 4dpf T3-treated zebrafish showing *lws1* expression superimposed with selected opsin transcripts.

A) Representative images displaying the *lws1* (magenta) channel overlaid with *sws1, sws2, rh2-1, rh2-2,* and *rho* label. No clear co-labeling of *lws1* with other opsins was observed in either wildtype or transgenic groups. Occasionally, some *rh2* positive cones overlapped with *lws1* labeling, although this was rare. Inset shows enlarged regions with individual channels separated to highlight the absence of overlap. Scale bar = 10μm.

A

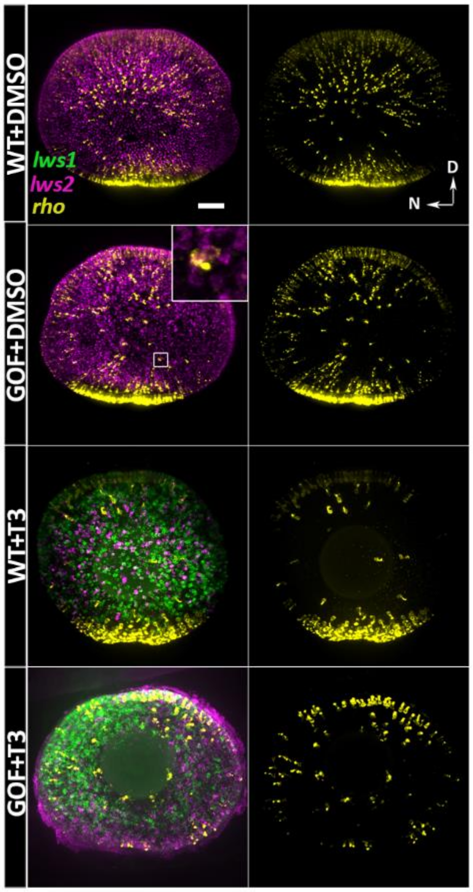

B

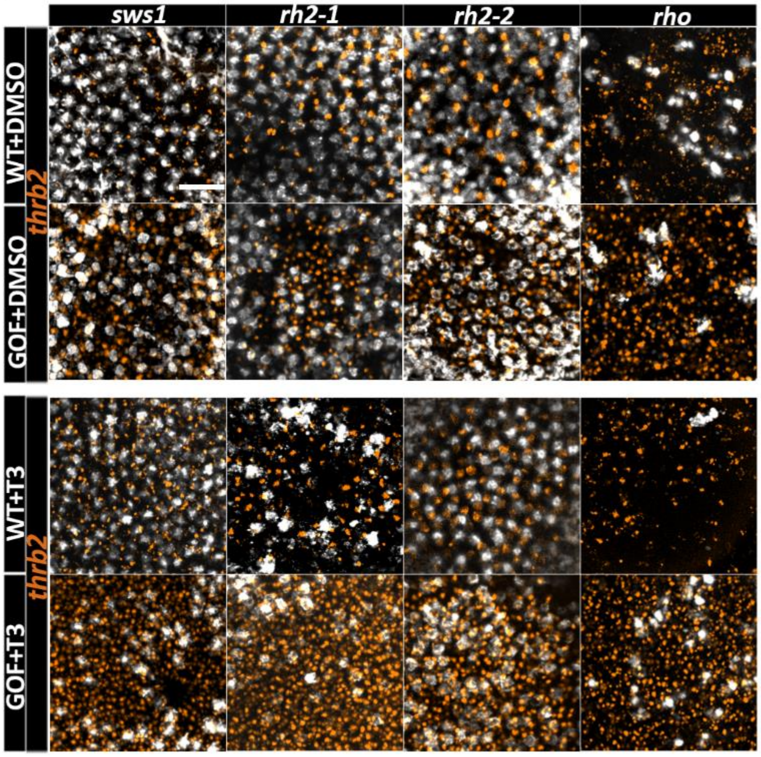

Supplemental Figure S3. Fluorescence *in situ* hybridization (HCR) analysis of opsin and *trβ2* expression in 4 and 6 dpf zebrafish. **A)** Confocal images of 6dpf wildtype and *trβ2* GOF transgenic fish, showing *rho* expression overlaid with *lws1* and *lws2*. No significant co-labeling of *rho* with either *lws1* or *lws2* was observed, except for rare instances where *rho* co-labeled with lws2 in the *trβ2* GOF+DMSO group (inset). The second column in A demonstrates a reduction in the number of *rho*+ cells in the presence of elevated *trβ2* receptor levels. B) Whole-mounted retinas from 4dpf wildtype and transgenic larvae, showing the pattern of *trβ2* mRNA expression relative to non *lws*+ cones under DMSO and T3-treated conditions. In wildtype fish, *trβ2* was not co-expressed with other opsin subtypes in individual cones (WT+DMSO and WT+T3 rows). However, in both transgenic groups, *trβ2* co-expression increased, particularly in the T3-treated group (GOF+T3). Scale bars in A=50μm and B=10μm.

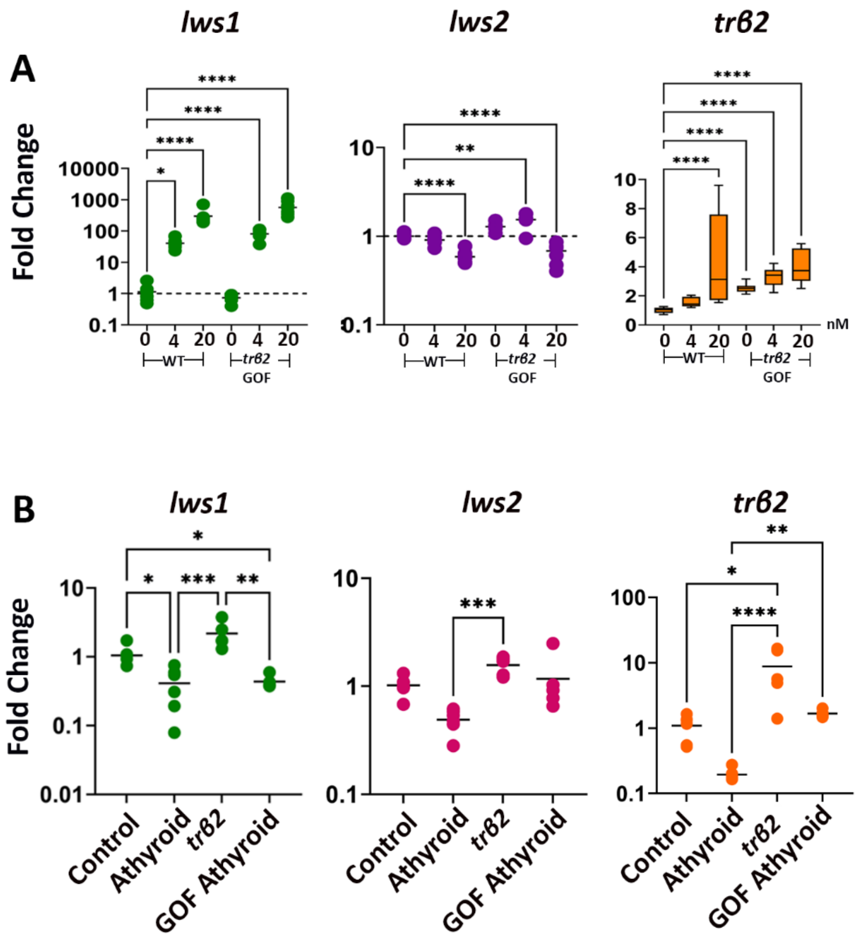

Supplemental Figure S4. **RT-qPCR analysis of wildtype and transgenic larvae showing differential regulation of *lws1* and *lws2***.

A) Scatter and box-and-whisker plots represent fold change in transcript expression (2^-ΔΔCT) following 48 hours of 4nM T3 treatment. Each point represents a biological replicate (n ≥ 6). B) RT-qPCR analysis of *lws1*, *lws2*, and *trβ2* transcripts from larvae generated by crossing Tg(*crx:mYFP-2a-trβ2*) with homozygous Tg(*tg:nVenus-2a-nfsB*) $^{wp.rt8}$ transgenic line. Larvae were treated with metronidazole or DMSO (control) for 24 hours and then sorted for thyroid gland ablation at 6 dpf. P-values were calculated using Kruskal-Wallis one-way ANOVA with post-hoc testing adjusted by Dunn's correction. Statistical significance is denoted as *P < 0.05, **P < 0.01, ***P < 0.001, ****P < 0.0001. hpt: hours post treatment. D= Dorsal N= Nasal.

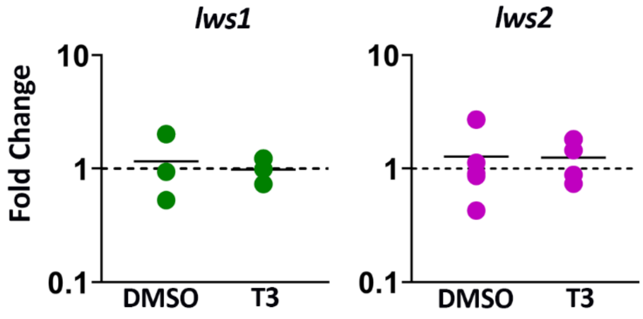

Supplemental Fig S5. Loss of *trβ* gene function abolishes T3 regulation of *lws1* and *lws2.*

Scatter plots show relative fold-change expression ($2^{-\Delta\Delta CT}$) for *lws1* and *lws2* in response to T3 in the *trβ* mutants. Each point represents a biological replicate ($n \geq 6$). No significant differences were observed between treatments, with transcripts undetectable in 3 additional replicates, demonstrating that a functional *trβ* gene is required for T3-mediated regulation of LWS opsins.
